# Age Differences in Cardiopulmonary Exercise Testing Parameters in Heart Failure with Reduced Ejection Fraction

**DOI:** 10.3390/medicina59091685

**Published:** 2023-09-20

**Authors:** Pedro Garcia Brás, António Valentim Gonçalves, João Ferreira Reis, Rita Ilhão Moreira, Tiago Pereira-da-Silva, Pedro Rio, Ana Teresa Timóteo, Sofia Silva, Rui M. Soares, Rui Cruz Ferreira

**Affiliations:** 1 Cardiology Department, Santa Marta Hospital, Central Lisbon Hospital University Center, 1169-024 Lisbon, Portugal; 2 NOVA Medical School, Faculdade de Ciências Médicas (NMS|FCM), 1169-056 Lisbon, Portugal

**Keywords:** heart failure with reduced ejection fraction, cardiopulmonary exercise testing, peak oxygen consumption, VE/VCO_2_ slope, age, heart transplantation

## Abstract

*Background and Objectives*: Cardiopulmonary exercise testing (CPET) is a cornerstone of risk stratification in heart failure with reduced ejection fraction (HFrEF). However, there is a paucity of evidence on its predictive power in older patients. The aim of this study was to evaluate the prognostic power of current heart transplantation (HTx) listing criteria in HFrEF stratified according to age groups. *Materials and Methods*: Consecutive patients with HFrEF undergoing CPET between 2009 and 2018 were followed-up for cardiac death and urgent HTx. *Results*: CPET was performed in 458 patients with HFrEF. The composite endpoint occurred in 16.8% of patients ≤50 years vs. 14.1% of patients ≥50 years in a 36-month follow-up. Peak VO_2_ (pVO_2_), VE/VCO_2_ slope and percentage of predicted pVO_2_ were strong independent predictors of outcomes. The International Society for Heart and Lung Transplantation thresholds of pVO_2_ ≤ 12 mL/kg/min (≤14 if intolerant to β-blockers), VE/VCO_2_ slope > 35 and percentage of predicted pVO_2_ ≤ 50% presented a higher overall diagnostic effectiveness in younger patients (≤50 years). Specific thresholds for each age subgroup outperformed the traditional cut-offs. *Conclusions*: Personalized age-specific thresholds may contribute to an accurate risk stratification in HFrEF. Further studies are needed to address the gap in evidence between younger and older patients.

## 1. Introduction

Cardiopulmonary exercise testing (CPET) is a crucial exam in the risk stratification of patients with heart failure (HF) with reduced ejection fraction (HFrEF), including in the decision for heart transplantation (HTx) listing [1,2]. Peak oxygen consumption (pVO_2_) [3,4,5] and the minute ventilation–carbon dioxide production ratio (VE/VCO_2_ slope) [3,5,6] are strong predictors of HF outcomes in patients with HFrEF. In the 2016 International Society for Heart and Lung Transplantation (ISHLT) listing criteria for HTx [7], a pVO_2_ threshold of ≤12 mL/kg/min (in patients taking β-blockers) [8], and a threshold of ≤14 mL/kg/min (in patients intolerant of β-blockers) are the recommended values. A VE/VCO_2_ slope of >35 and a percentage of predicted pVO_2_ ≤50% may be considered in conjunction with pVO_2_ for risk stratification in young patients (<50 years) [7]. However, the evidence for these thresholds stems from trials enrolling primarily middle-aged male patients, with a mean age of around 59 years [2,3,5].

Aging is associated with physiological changes and reduced functional capacity, characterized by a decline of approximately 0.4–0.5 mL/kg/min of pVO_2_ per decade [9,10], with a similar rate of decline between sexes [1], and an increase in VE/VCO_2_ slope [11]. In addition, there is a higher prevalence of chronotropic incompetence, lower diastolic compliance, lower skeletal muscle mass, decreased capillary-to-muscle-fiber ratio, and metabolic changes in skeletal muscle [12]. The vast majority of trials on risk assessment with CPET in HFrEF enrolled middle-aged patients [13], and therefore, their discriminative power should be interpreted with caution in younger or elderly patients [1], especially in the latter, as a substantial number of patients are excluded from trials due to comorbidities limiting use of CPET or disease severity [10]. Nevertheless, the predictive power of CPET in elderly patients with HFrEF was shown in large studies [14,15,16].

Although the ISHLT guidelines [7] recommend alternative CPET approaches for younger patients (<50 years), the evidence of these parameters in young patients is poorly supported (class IIa, level of evidence B), as unbiased data are not currently available [1].

Our aim was to assess the prognostic difference in ISHLT-recommended CPET thresholds for HTx listing in patients with HFrEF between patients younger than 50 years of age and patients over 50 years of age.

## 2. Materials and Methods

### 2.1. Study Population

A single-center retrospective analysis of a prospectively collected database was performed, including patients who underwent CPET from January 2009 to December 2018. We enrolled consecutive HFrEF patients with a left ventricular ejection fraction (LVEF) of less than 40%, in New York Heart Association (NYHA) class II or III, who were referred to the HF team to assess the indication for HTx or mechanical circulatory support (MCS).

### 2.2. Study Protocol

The study included an assessment of the patients’ clinical data, including NYHA class, medical therapy, HF etiology, comorbidities, Heart Failure Survival Score (HFSS), cardiac implanted electronic devices, laboratory testing, ECG, echocardiographic data, and CPET parameters.

### 2.3. Exclusion Criteria

1. Younger than 18 years;
2. Planned or recent coronary revascularization or cardiac surgery;
3. Exercise-limiting comorbidities (cerebrovascular disease, severe peripheral vascular disease, or orthopedic disorders);
4. Previous HTx;
5. Elective HTx during the follow-up period;
6. Submaximal CPET (defined as one with a peak RER of ≤1.05 [7]);
7. Lost to follow-up.

### 2.4. Cardiorespiratory Exercise Testing

A GE Marquette Series 2000 treadmill employing the modified Bruce protocol was used to perform maximal symptom-limited CPET. Calibration of the equipment was performed before each exercise test. A SensorMedics Vmax 229 gas analyzer was used to acquire the VE, VO_2_, and VCO_2_ values. The heart rate (HRt) was measured by continuous ECG. A sphygmomanometer was used to obtain blood pressure (BP) values, and pulse oximetry was employed to monitor O_2_ saturation. A cardiopulmonary exercise test was defined as maximal when the respiratory exchange ratio (RER) was over 1.05, as recommended by the ISHLT [7]. The highest 30 s average achieved during exercise was used to define the pVO_2_, which was normalized for body mass. With the data acquired during exercise, least squares linear regression was used to calculate the VE/VCO_2_ slope. The minimum VE/VO_2_ was used to define the cardiorespiratory optimal point (COP). The partial pressure of end-tidal carbon dioxide (PetCO_2_) was measured before exercise and at the anaerobic threshold (AT). Dividing the pVO_2_ by the maximum HRt during exercise was performed to calculate the peak O_2_ pulse. Ventilatory power was determined by dividing the peak systolic BP by the VE/VCO_2_ slope. Circulatory power was calculated as the product of pVO_2_ and peak systolic BP. The difference between the maximum HRt with exercise and the resting HRt was used to determine the HRt reserve. The HRt recovery in the first minute after exercise was determined by the difference between the maximum HRt achieved and the HRt one minute into recovery.

### 2.5. Primary Endpoint

Patients were followed up for 36 months. The primary endpoint was defined as a composite of cardiovascular death or urgent HTx during an unplanned HF hospitalization. The patients’ medical data were collected from the outpatient clinic digital records and from inpatient visits.

### 2.6. Statistical Analysis

Statistical analyses were performed to compare patients according to age: under 50 years of age vs. over 50 years of age. Statistical analysis was performed using the Statistical Package for the Social Sciences (SPSS) for Windows, Version 23.0 (IBM Corp, Armonk, NY, USA). Point estimates and 95% CI were presented for all mean estimates.

For categorical variables, descriptive statistics were presented as the absolute frequency (number) and relative frequency (percentage). Normally distributed continuous variables were reported as the mean (and standard deviation), and non-normally distributed continuous variables were presented as the median (and interquartile range [IQR]). The Kolmogorov–Smirnov test and visual analysis of the histogram were used to test normality assumptions.

Categorical variables were compared with Pearson’s chi-squared test. Continuous variables we compared using the Student’s *T*-test (for normally distributed variables) and the Mann–Whitney U test (for non-normally distributed variables).

A Cox proportional hazards regression model was used to assess the association of CPET parameters with the composite endpoint. In the univariate analysis, the variables with a *p*-value < 0.200 were considered in the multivariate analysis to adjust for potential confounders. The hazard ratios (HR) were determined for the total cohort and for each age subgroup. The results were presented as adjusted HR and 95% confidence interval (CI).

The sensitivity and specificity of each CPET parameter in predicting the composite endpoint were explored by receiver operating characteristic (ROC) curves according to the ISHLT thresholds [7]: pVO_2_ ≤ 12 mL/Kg/min (pVO_2_ ≤ 14 mL/Kg/min if intolerant to β-blockers), VE/VCO_2_ slope >35 and percentage of predicted pVO_2_ ≤ 50%. The cut-off value combining the highest sum of sensitivity and specificity was identified with the Youden index (*J*). The DeLong et al. test [17] was used to assess the significance of the difference between the areas under the curves (AUC) from each group’s ROC curves.

Moreover, the event-free survival rate was determined with the Kaplan–Meier analysis. A log-rank test was performed to compare the age subgroups according to the ISHLT-guideline-recommended cut-off values for pVO_2_, VE/VCO_2_ slope, and percentage of predicted pVO_2_ [7], and according to the alternative thresholds. A significance level of α = 5% was considered in all statistical analysis.

## 3. Results

### 3.1. Baseline Characteristics

**Figure 1:**
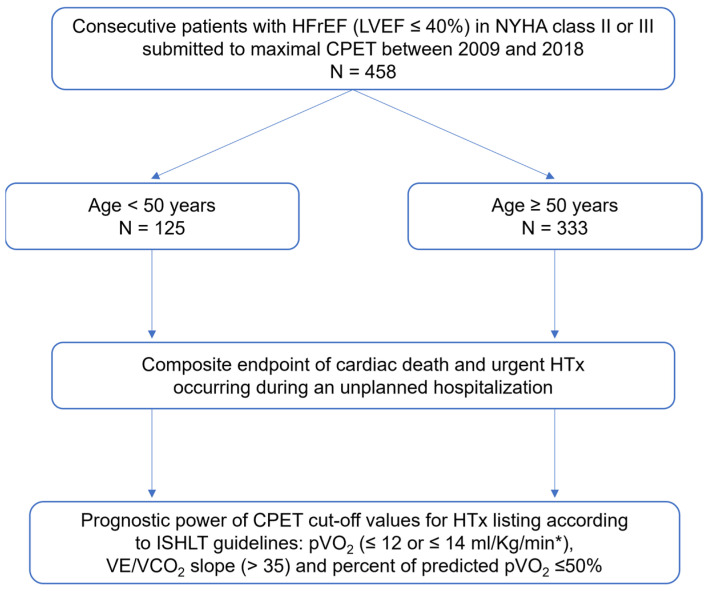
Study flowchart. * in patients intolerant to β-blockers. HFrEF: Heart failure with reduced ejection fraction; CPET: Cardiopulmonary exercise test; HTx: Heart transplantation; LVEF: Left ventricular ejection fraction; NYHA: New York Heart Association; ISHLT: International Society for Heart and Lung Transplantation; pVO_2_: Peak oxygen consumption; VE/VCO_2_ slope: Minute ventilation–carbon dioxide production ratio.

A total of 458 patients undergoing maximal CPET were included. The study population flowchart is presented in Figure 1. The mean age was 56 ± 12 years, with 27.3% of patients younger than 50 years of age. 79% were males, 57% with ischemic HF etiology, 76% in NYHA class II, 24% in NYHA class III, 24% with atrial fibrillation (AF), and a mean LVEF of 29.7 ± 8.0%. Moreover, 79% of patients were taking angiotensin-converting-enzyme inhibitors (ACEi) or angiotensin receptor blockers (ARBs), and 17% were taking sacubitril/valsartan. Most patients (86%) were on β-blockers and 73% were on mineralocorticoid receptor antagonists (MRAs). In addition, 10% of patients were on sodium-glucose cotransporter-2 inhibitors (SGLT2i). The majority of patients (64%) had an ICD, out of which 22% had a cardiac resynchronization therapy device (CRT-D). Furthermore, the mean HFSS was 8.6 ± 1.10, with no difference between subgroups. Patients older than 50 years had a higher prevalence of diabetes mellitus, chronic kidney disease, and AF. Patients under 50 years of age showed a higher pVO_2_, a lower percentage of predicted pVO_2_, a higher VE/VCO_2_ slope, and a higher circulatory power compared with patients over 50 years of age. The mean respiratory exchange ratio (RER) was 1.14 ± 0.07. The baseline characteristics of both groups and the CPET parameters are presented in Table 1.

### 3.2. Composite Endpoint

The composite endpoint occurred in 68 (14.8%) patients, with cardiac death in 54 patients and urgent HTx in 14 patients in a 36-month follow-up (Table 2). No patients required urgent MCS. The composite endpoint occurred in 16.8% of patients ≤50 years vs. 14.1% of patients ≥50 years, with no statistically significant difference between age groups. Patients under 50 years of age had a higher rate of urgent HTx (5.6% vs. 2.1%, *p* = 0.002), as shown in Table 2.

### 3.3. Relationship between Cardiopulmonary Exercise Test Prognostic Parameters and the Primary Endpoint

In a multivariable time-dependent Cox regression model, pVO_2_ (adjusted HR 0.856, *p* < 0.001), VE/VCO_2_ slope (adjusted HR 1.064, *p* < 0.001), and the percentage of predicted pVO_2_ (adjusted HR 0.955, *p* < 0.001) correlated with the composite endpoint in a 36-month follow-up, regardless of age group. The univariable and multivariable analyses are presented in Table 3. In the multivariable analysis, these correlations were independent of sex, age, LVEF, estimated glomerular filtration rate, and smoking, body mass index, and diabetes mellitus. Most of the remaining CPET parameters were not associated with the composite endpoint in the multivariable model. The peak O_2_ pulse was a predictor of outcomes in patients over 50 years of age. The circulatory power, ventilatory power, and PetCO_2_ at AT were associated with the composite endpoint in both age subgroups (Table 3).

**Figure 2:**
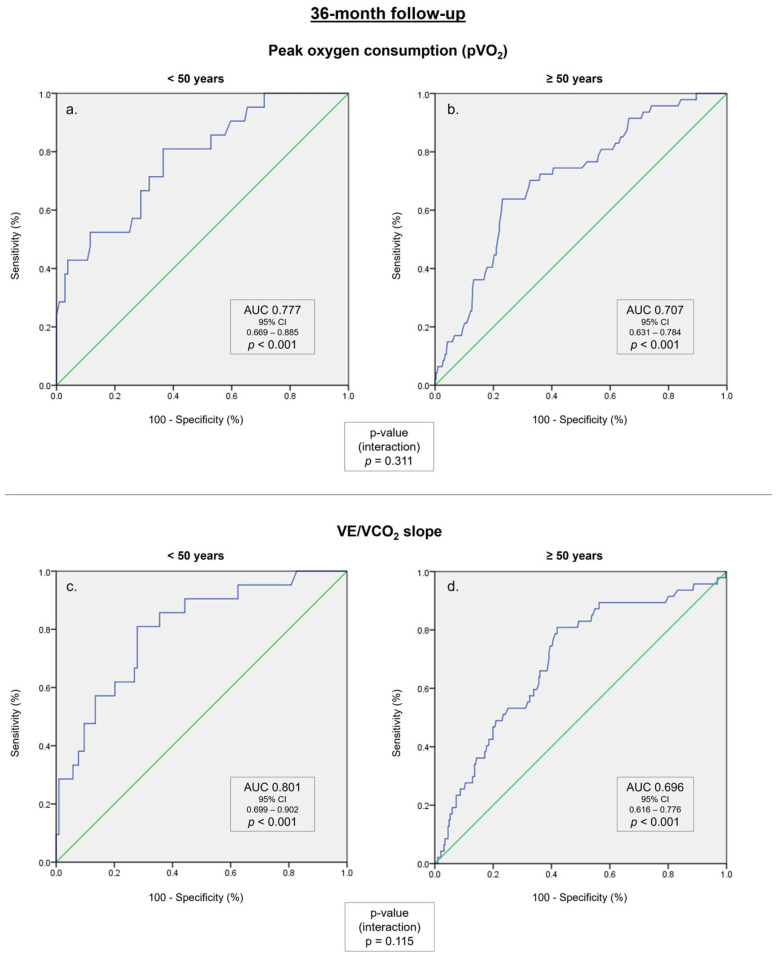
ROC curves for the composite endpoint in a 36-month follow up are presented in blue. The diagonal reference line is presented in green. (**a**) Peak oxygen consumption (pVO_2_) in patients under 50 years old. (**b**) pVO_2_ in patients over 50 years old. (**c**) Minute ventilation–carbon dioxide production ratio (VE/VCO_2_ slope) in patients under 50 years old. (**d**) VE/VCO_2_ slope in patients over 50 years old.

In a ROC curve analysis (Figure 2, Supplementary Figure S1), the pVO_2_, VE/VCO_2_ slope, and the percentage of predicted pVO_2_ were associated with the composite endpoint in a 36-month follow-up in both subgroups. In the age subgroup analysis (Table 4), the correlations between traditional CPET parameters and the composite endpoint were numerically higher in patients under 50 years of age compared with patients over 50 years of age, either for pVO_2_ (AUC 0.777 vs. AUC 0.707, *p* = 0.311), for VE/VCO_2_ slope (AUC 0.818 vs. AUC 0.703, *p* = 0.060), or for the percentage of predicted pVO_2_ (AUC 0.801 vs. AUC 0.703, *p* = 0.115).

The circulatory power showed a similar AUC to the traditional CPET parameters in patients over 50 years of age (AUC 0.709 vs. AUC 0.707, *p* = 0.526), with no statistically significant differences in predictive power. Although other CPET parameters, including peak O_2_ pulse, PetCO_2_ at rest, PetCO_2_ at AT, COP, and ventilatory power, were associated with the composite endpoint, their predictive power was lower than the pVO_2_, the VE/VCO_2_ slope, or the percentage of predicted pVO_2_ (Table 4).

### 3.4. ISHL Thresholds for HTx Listing

**Figure 3:**
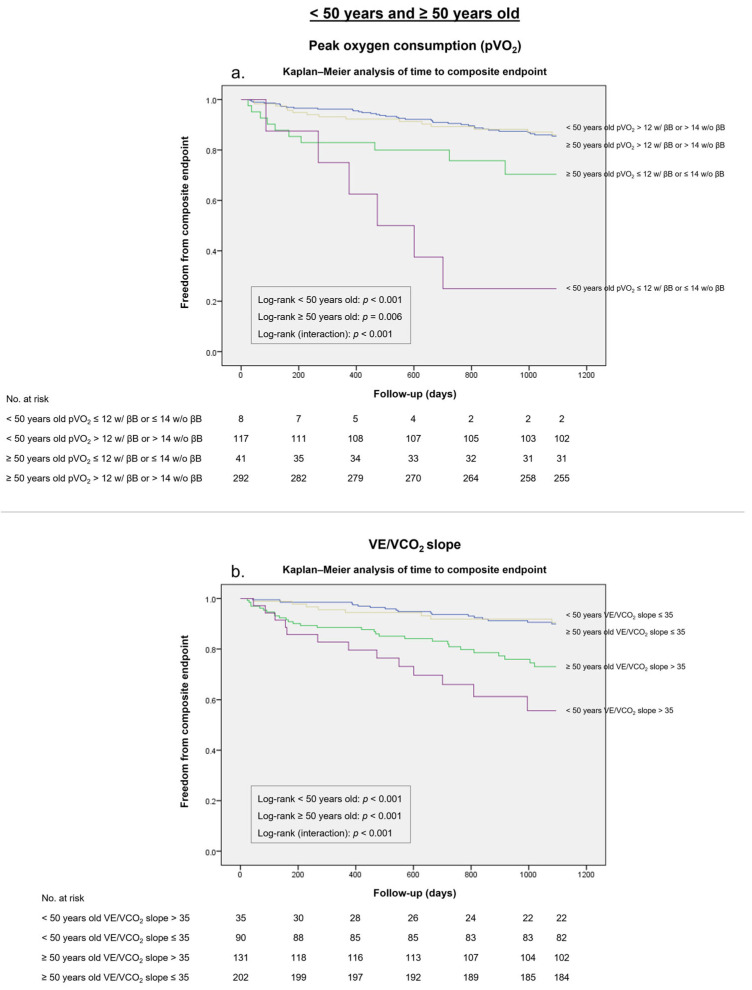
Kaplan–Meier survival analysis for the composite endpoint in a 36-month follow-up stratified according to the International Society for Heart and Lung Transplantation (ISHLT) guidelines in patients under 50 years old and patients over 50 years old. (**a**) Peak oxygen consumption (pVO_2_) ≤12 mL/Kg/min (≤14 mL/kg/min if intolerant to β-blockers [βB]). (**b**) Minute ventilation–carbon dioxide production ratio (VE/VCO_2_ slope) of >35.

A pVO_2_ of ≤12 mL/kg/min (or of ≤14 mL/kg/min if intolerant to β-blockers) presented a higher Youden index in patients under 50 years of age (*J* 0.27 vs. *J* 0.10), with a specificity of 98% and a sensitivity of 29%, compared to patients over 50 years of age, with a specificity of 89% and a sensitivity of 21% in patients over 50 years of age (Table 5). This threshold was an accurate predictor of worse outcomes at 36 months in the survival analysis (Figure 3a).

Likewise, the VE/VCO_2_ slope cut-off of >35 presented a specificity of 80% and a sensitivity of 62% for the composite endpoint in patients under 50 years old, with a specificity of 64% and a sensitivity of 62% in patients over 50 years old. The Youden index associated with this threshold was higher in patients under 50 years old compared with patients over 50 years old (*J* 0.42 vs. *J* 0.26). The survival curves (Figure 3b) showed that this threshold was an accurate predictor of the composite endpoint in both age subgroups.

Furthermore, the percentage of predicted pVO_2_ ≤ 50% threshold had a specificity of 71% and a sensitivity of 62% for the composite endpoint in patients under 50 years of age, with a slightly higher Youden index than patients over 50 years of age (*J* 0.33 vs. *J* 0.30), in which this cut-off showed a specificity of 83% and a sensitivity of 47% (Table 5). The Kaplan–Meier survival analysis (Supplementary Figure S2a) revealed that the percentage of predicted pVO_2_ ≤ 50% threshold was a strong predictor of the composite endpoint in both patients under 50 years old (log-rank *p* < 0.001) and patients over 50 years old (log-rank *p* = 0.006).

### 3.5. Alternative pVO_2_ and VE/VCO_2_ Slope Cut-Offs

**Figure 4:**
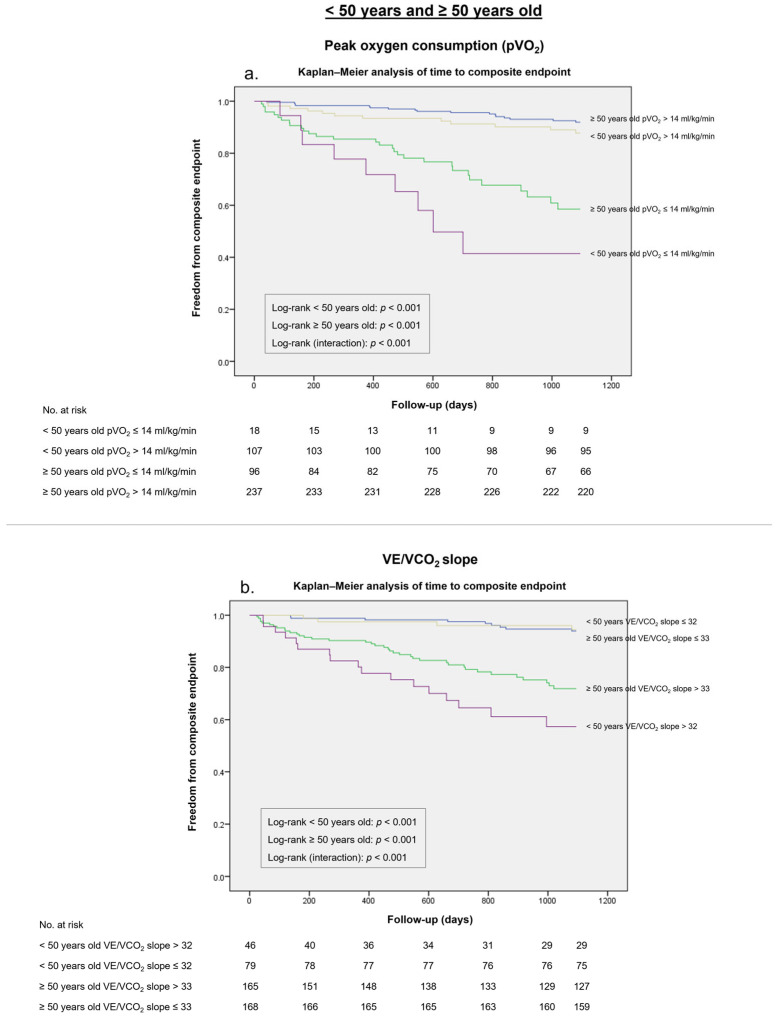
Kaplan–Meier analysis for the composite endpoint in patients under 50 years old and patients over 50 years old stratified according to (**a**) Peak oxygen consumption (pVO_2_) of ≤14 mL/Kg/min. (**b**) Minute ventilation–carbon dioxide production ratio (VE/VCO_2_ slope) of >32 and >33, respectively.

A pVO_2_ ≤ 14 mL/kg/min threshold presented a specificity of 89% and a sensitivity of 52% for the composite endpoint in patients under 50 years of age, and a specificity of 77% and a sensitivity of 64% in patients over 50 years of age (Table 5). This threshold’s overall diagnostic effectiveness was higher for patients under 50 years old (*J* 0.41 vs. *J* 0.27) and particularly for patients over 50 years old (*J* 0.41 vs. *J* 0.10), which was confirmed in the survival analysis (log-rank *p* < 0.001) (Figure 4a).

A VE/VCO_2_ slope >32 threshold yielded a higher Youden index in patients under 50 years old in comparison with the traditional cut-off (*J* 0.53 vs. *J* 0.42), while a VE/VCO_2_ slope >33 presented a slightly higher Youden index compared with the >35 cut-off (*J* 0.39 vs. *J* 0.35). Likewise, both VE/VCO_2_ slope >32 and >33 cut-offs were accurate discriminators of the composite endpoint at 36 months (log-rank *p* < 0.001) (Figure 4b).

Moreover, a percentage of predicted pVO_2_ of ≤58% and of ≤56% presented a higher Youden index in patients under 50 years old (*J* 0.50 vs. *J* 0.33) and in patients over 50 years old (*J* 0.37 vs. *J* 0.30), respectively, compared with the traditional ≤55% cut-off. Similarly, the significant discriminative power of these proposed thresholds is illustrated in the survival analysis (log-rank *p* < 0.001) (Supplementary Figure S2b).

## 4. Discussion

The main conclusion of our paper was that, while pVO_2_, VE/VCO_2_ slope and percentage of predicted pVO_2_ presented a significant prognostic power for major HF outcomes both in younger and older patients, the ISHLT-recommended thresholds for pVO_2_ (≤12 mL/kg/min, or ≤14 mL/kg/min if intolerant to β-blockers), VE/VCO_2_ slope (>35), and percentage of predicted pVO_2_ ≤ 55% showed a significantly higher overall diagnostic effectiveness in young patients in comparison with patients over 50 years of age.

In addition, we evaluated the predictive power of other CPET parameters and assessed alternative thresholds for pVO_2_, for VE/VCO_2_ slope, and for the percentage of predicted pVO_2_, which may contribute to a higher prognostic effectiveness in patient selection for HTx listing in different age groups.

HFrEF is a multifactorial entity in older patients, encompassing several factors such as cardiovascular changes due to aging, with an age-related decline of pVO_2_ [9,10], lifelong lifestyle habits, and an increased prevalence of concomitant heart disease, such as ischemic heart disease, hypertensive heart disease, valvular disease, or AF. Comorbidities (e.g., chronic obstructive pulmonary disease, chronic kidney disease, or orthopedic disorders) are frequently present in this population [16]. A trial by Kim et al. [18] showed that CPET was a safe and useful modality for the assessment of exercise capacity in an elderly population with comorbidities. However, risk stratification in this group of patients may be challenging, as data are scarce regarding the discriminative power of the available prognostic models in older patients [10,19].

In our cohort, patients over the age of 50 presented a substantially lower pVO_2_, a lower percentage of predicted pVO_2_, and a higher VE/VCO_2_ slope. Our results showed that younger and older patients had similar outcomes in a 3-year follow-up, although there was a higher rate of urgent HTx in younger patients. A possible explanation for this finding is a higher prevalence of comorbidities and the reported inferior outcomes of HTx in older patients. In a trial by Goldstein et al., patients over the age of 70 benefited from HTx, suffering less allograft rejection but presenting a higher mortality rate than younger patients [20]. Based on the current evidence, ISHLT guidelines recommend HTx only in carefully selected cases in patients over 70 years of age [7]. Nevertheless, these guidelines state that local policies should define the upper age limit for eligibility to transplant in the context of local organ availability to maintain acceptable transplant outcomes and allow for transplantation of all listed patients.

In our study, traditional CPET parameters showed a numerically inferior predictive power in older patients compared with patients under 50 years of age, albeit with no statistical difference between groups. The recommended thresholds for pVO_2_ (≤12 mL/kg/min, or ≤14 mL/kg/min if intolerant to β-blockers) and VE/VCO_2_ slope (>35) presented a higher diagnostic effectiveness in young patients in comparison with patients over 50 years of age.

Evidence on risk stratification of elderly patients has proven that CPET is safe and reproducible, and accurately predicts prognosis [10], as pVO_2_ and VE/VCO_2_ slope were the strongest independent predictors of prognosis in this population [14,15,16,21,22,23,24]. A trial by Arena et al. [15] comparing patients according to age subgroups showed that pVO_2_ was a more robust prognostic marker in the ≤45 and ≥66-year subgroups, while the percentage of predicted pVO_2_ provided the highest discriminative power in patients aged 46 to 65.

Importantly, the current definition of normal VE/VCO_2_ slope values is inadequate, as it was defined according to data stemming from small groups of patients with a particularly limited number of women and elderly patients [25,26]. In a recent trial by the metabolic exercise cardiac kidney index (MECKI) research group [27], the authors attempted to update the predictive role of the VE/VCO_2_ slope for the sex and age of HFrEF patients [19]. In the reported findings, the percentage of predicted VE/VCO_2_ slope showed a higher prognostic power than the absolute value of VE/VCO_2_ slope for HF prognostication in patients with severe HfrEF (with low pVO_2_). Thus, the authors highlighted that VE/VCO_2_ slope expressed as the percentage of predicted value can contribute to a stronger prognostic prediction of high-risk HFrEF patients.

Our findings show that alternative cut-offs for pVO_2_ (≤14 mL/kg/min, including patients on β-blockers), VE/VCO_2_ slope (>33), and percentage of predicted pVO_2_ (≤56%) presented a higher diagnostic effectiveness in the older subgroup compared with the traditional ISHLT thresholds, and therefore may be an alternative option to improve risk stratification in older patients. In patients under 50 years old, the strongest predictor of HF outcomes was the percentage of predicted pVO_2_. Similarly, a threshold of ≤58% had a higher diagnostic effectiveness in this subgroup than the ISHLT-recommended cut-off of ≤50%.

Circulatory power (pVO_2_ × peak systolic BP) is a non-invasively determined surrogate of cardiac power [28]. In a study by Lala et al. [29], circulatory power was the strongest predictor of MCS implantation, transplantation, or death at 1 year in a contemporary ambulatory advanced HFrEF population, suggesting that circulatory power should be reviewed as an integral part of the CPET output. In a recent trial by Lewis et al. [30,31], circulatory power showed a greater discriminative ability to predict HF outcomes compared with O_2_ pulse and pVO_2_. In our cohort, circulatory power was found to be a strong predictor of the composite endpoint, particularly in patients over 50 years of age. However, further evidence is needed to evaluate the discriminative power of circulatory power for HF outcomes in older patients.

In older patients, VE, VO_2_, and VCO_2_ present slower recovery kinetics compared with younger patients, partly due to a delay in the removal of exercise-induced CO_2_ attributed to an age-related decrease in central or peripheral CO_2_ chemosensitivity [32]. As recovery oxygen kinetics are independent of the level of exercise achieved, their prognostic role has been studied in elderly patients [33]. In a retrospective study by Fortin et al. [34], recovery oxygen kinetics provided an incremental prognostic value over using only oxygen kinetics during CPET. Moreover, recovery VO_2_ was shown to be a stronger predictor of mortality, HTx, or MCS than pVO_2_ in patients with severe HF, and thus may be an alternative parameter for risk stratification in HFrEF [34].

The evolving management of HFrEF should also be considered, as our study examined patients who underwent CPET from 2009 to 2018, spanning a wide array of increasingly diverse HFrEF therapies. In the last decade, the expanding role of guideline-directed medical therapy, including newly developed drug classes and cardiovascular procedures such as cardiac resynchronization therapy, AF catheter ablation, or mitral transcatheter edge-to-edge repair, has led to a new paradigm in HFrEF management with a substantial improvement in cardiovascular outcomes [35,36].

A recent study [37] evaluated how the prognostic thresholds of pVO_2_ and of VE/VCO_2_ slope have changed between 1993 and 2015 in parallel with the improvement of HFrEF prognosis. The pVO_2_ and VE/VCO_2_ slope cut-offs associated with a substantial risk of HF events progressively decreased and increased, respectively. The authors concluded that the prognostic cut-offs of pVO_2_ and VE/VCO_2_ slope should be updated whenever the HFrEF prognosis improves. The majority of patients in every subgroup of our study were on guideline-directed medical therapy, including β-blockers and mineralocorticoid receptor antagonists. However, all patients in our study were included between 2009 and 2018, when SGLT2i were not considered guideline-directed medical therapy for patients without diabetes mellitus; thus, only 10% of patients in our cohort were taking SGLT2i. Moreover, less than one-quarter of the patients in our study were taking sacubitril/valsartan. Therefore, the prognostic value of CPET parameters in older patients should also be examined in trials enrolling patients with contemporary HF management strategies [38,39,40], including a higher percentage of patients taking SGLT2i and sacubitril/valsartan.

Prognostic risk scores may also aid in the risk stratification of patients with HFrEF. A Seattle Heart Failure Model (SHFM) [41,42] <80% or a medium-to high-risk HFSS [43] are complementary criteria in the ISHTL recommendations to consider HTx listing [7]. Combining risk scores with CPET parameters may also assist in HFrEF risk stratification, as combining pVO_2_ with the SHFM was shown to improve prognostic accuracy in patients with intermediate risk [44]. Both the HFSS and the MECKI score [45] incorporate CPET variables, namely pVO_2_ and percentage of predicted pVO_2_, respectively, to characterize the patient’s functional status. Furthermore, the MECKI score was higher in older patients, but its predictive power was independent of patient age, showing a similar prognostic value across age subgroups. Therefore, the MECKI score may also assist in the risk stratification of older patients with HFrEF [16,19].

### Limitations

Firstly, our study was a retrospective analysis of a prospectively collected database with a small sample, including patients from a single center, and thus, our findings would need confirmation in prospective, randomized trials in multiple centers for validation. Furthermore, while 73% of our cohort was over 50 years old, this subgroup’s mean age was 61 ± 8 years. Therefore, our results may not be applicable to elderly patients with HFrEF.

Secondly, although the subgroups in our cohort were unmatched, patients were enrolled consecutively, thus compensating for the lack of randomization. Moreover, most baseline characteristics were similar in both age subgroups, including NYHA class, body mass index, LVEF, LV dimensions, right ventricular dysfunction, mitral regurgitation, HFSS, and ICD/CRT implantation. As expected [10], older patients had a higher prevalence of comorbidities, including AF, chronic kidney disease, and diabetes.

Thirdly, the alternative pVO_2_ thresholds of ≤14 mL/kg/min and ≤15 mL/kg/min were evaluated in patients in every subgroup, regardless of whether they were taking β-blockers. As most patients (86%) were on β-blockers, our paper did not have significant statistical power to estimate a pVO_2_ threshold for patients intolerant to β-blockers. Therefore, the alternative pVO_2_ thresholds may not be appropriate in this patient subgroup.

Our study only included patients who underwent maximal CPET. There is no current consensus on the peak RER threshold to define maximal exercise, especially in patients with HFrEF. Various thresholds were proposed, ranging from 1.0 to 1.10 [1,46,47,48]. In our study, as our aim was the assessment of risk stratification for HTx listing, we used the ISHLT definition for maximal CPET, enrolling patients with a peak RER over 1.05 [7]. Therefore, our findings and alternative thresholds may not be reproducible in a general HFrEF population, as a submaximal CPET was an exclusion criterion, and pVO_2_ has shown a lower predictive value in this subgroup of patients [49]. Furthermore, while a single cardiopulmonary exercise test was performed in our study, serial CPET may provide further insight into the risk stratification of HFrEF.

Lastly, HFrEF severity was lower in our cohort compared with that of other studies [50], as the pVO_2_ was higher and there was a lower rate of HF outcomes, especially urgent HTx. This may also affect the widespread application of our findings. All patients in our study were referred to the local HF team to assess whether advanced HF therapies were indicated, and consequently, our results may not be reproducible in a general HFrEF population with a higher comorbidity burden.

## 5. Conclusions

In patients with HFrEF undergoing CPET, the pVO_2_, VE/VCO_2_ slope, and the percent of predicted pVO_2_ were the strongest independent predictors of mortality. The ISHLT-guideline-established cut-off values for pVO_2_ and VE/VCO_2_ slope presented a significantly higher overall diagnostic effectiveness in patients under 50 years old. Alternative pVO_2_, VE/VCO_2_ slope, and percentage of predicted pVO_2_ thresholds for each age subgroup outperformed the traditional cut-offs. Our findings suggest that individualized age-specific thresholds may be necessary to guide HTx listing. Further trials are needed to address the gap in HFrEF risk stratification data between younger and older patients.

## Figures and Tables

**Table 1:** Baseline characteristics of the study population (*n* = 458).

	Overall (*n* = 458)	<50 Years (*n* = 125)	≥50 Years (*n* = 333)	*p*-Value
Clinical and demographic data				
Age (years)	56 ± 12	39 ± 8	61 ± 8	<0.001
Male sex (*n*, %)	363 (79)	94 (75)	269 (81)	0.189
Body mass index (kg/m^2^)	27.1 ± 4.3	26.8 ± 5.1	27.2 ± 3.9	0.348
Ischemic etiology (*n*, %)	261 (57)	62 (50)	199 (60)	0.073
ACEi/ARB (*n*, %)	361 (79)	93 (74)	268 (80)	0.183
ARNI (*n*, %)	80 (17)	28 (22)	52 (16)	0.149
β-blocker (*n*, %)	392 (86)	104 (83)	288 (86)	0.422
MRA (*n*, %)	336 (73)	93 (74)	243 (73)	0.624
iSGLT2 (*n*, %)	47 (10)	9 (7)	38 (11)	0.124
Digoxin (*n*, %)	129 (28)	38 (30)	91 (27)	0.560
Diabetes	104 (23)	11 (9)	93 (28)	<0.001
CKD (*n*, %)	145 (32)	16 (13)	129 (39)	<0.001
AF (*n*, %)	109 (24)	18 (14)	91 (27)	0.004
ICD * (*n*, %)	293 (64)	84 (67)	210 (63)	0.225
CRT (*n*, %)	102 (22)	31 (25)	71 (21)	0.246
NYHA class II	347 (76)	95 (76)	252 (76)	0.358
NYHA class III	111 (24)	30 (24)	81 (24)	0.358
HFSS	8.6 ± 1.1	8.9 ± 1.2	8.6 ± 1.1	0.088
Laboratory data				
eGFR, mL/min/1.73 m^2^	75.3 ± 29.2	92.4 ± 31.2	69.3 ± 25.9	<0.001
Sodium, mEq/L	138.0 ± 3.0	137.5 ± 2.7	138.2 ± 3.1	0.036
NT-proBNP, pg/mL	2196 ± 2101	2116 ± 2013	2228 ± 2214	0.764
Echocardiographic data				
LVEDD, mm/m^2^	67.4 ± 10.3	69.2 ± 10.7	66.8 ± 10.2	0.171
LVEF, %	29.7 ± 8.0	30.0 ± 8.1	29.5 ± 8.1	0.564
MR III–IV, %	67 (14)	23 (18)	44 (13)	0.435
RV dysfunction (*n*, %)	69 (15)	15 (12)	54 (16)	0.486
CPET parameters				
Peak RER	1.14 ± 0.07	1.14 ± 0.06	1.14 ± 0.07	0.916
Delta HRt during exercise	51 (37–68)	64 (41–85)	47 (35–62)	<0.001
HHR1	17 (11–27)	22 (14–34)	16 (10–24)	<0.001
pVO_2_, mL/kg/min	18.5 ± 5.8	21.5 ± 7.0	17.4 ± 4.8	<0.001
Percentage of predicted pVO_2_ (%)	63.8 ± 18.7	60.3 ± 18.7	65.1 ± 18.5	0.014
VE/VCO_2_ slope	33.9 ± 9.6	32.3 ± 10.5	34.6 ± 9.2	0.030
pVO_2_, mL/kg/min at AT	13.6 ± 4.6	14.4 ± 5.4	13.4 ± 4.2	0.857
O_2_ pulse, mL/kg/beat	0.14 ± 0.06	0.15 ± 0.03	0.14 ± 0.07	0.380
Circulatory power, mmHg.mL/kg/min	2883 ± 1543	3288 ± 1467	2730 ± 1545	0.001
Ventilatory power, mmHg	4.8 ± 1.7	5.0 ± 1.7	4.7 ± 1.6	0.073
COP	28.9 ± 7.2	27.9 ± 8.7	29.2 ± 6.6	0.393
PetCO_2_ at rest, mmHg	33.6 ± 4.8	34.3 ± 5.1	33.3 ± 4.7	0.080
PetCO_2_ at AT, mmHg	36.8 ± 6.0	38.2 ± 6.4	36.3 ± 5.8	0.006

* including CRT-D; Values are presented as mean ± standard deviation or median (interquartile range). ARNI: Angiotensin receptor neprilysin inhibitors; AT: Anaerobic threshold; BB: Beta-blockers; BMI: Body mass index; COP: Cardiorespiratory optimal point; MRA: Mineralocorticoid receptor antagonists; CPET: Cardiopulmonary exercise test; ACEi: Angiotensin-converting enzyme inhibitors; AF: Atrial fibrillation; LVEDD: Left ventricular end-diastolic diameter; HFSS: Heart Failure Survival Score; HRR1: Heart rate recovery in the first minute after finishing CPET; HRt: Heart rate; ICD: Implantable cardioverter-defibrillator; CRT: Cardiac resynchronization therapy; LVEF: Left ventricular ejection fraction; CKD: Chronic kidney disease; ARB: Angiotensin receptor blockers; PetCO_2_: Partial pressure of end-tidal carbon dioxide; pVO_2_: Peak oxygen consumption; MR: Mitral regurgitation; RER: Respiratory exchange ratio; RV: Right ventricular; VE/VCO_2_ slope: Minute ventilation–carbon dioxide production ratio.

**Table 2:** Adverse events at 36-month follow-up.

	Overall (*n* = 458)	<50 Years (*n* = 125)	≥50 Years (*n* = 333)	*p*-Value
Combined primary endpoint (*n*, %)	68 (14.8%)	21 (16.8%)	47 (14.1%)	0.464
Total mortality (*n*, %)	67 (14.6%)	15 (12%)	52 (15.6%)	0.121
Cardiac mortality (*n*, %)	54 (11.8%)	14 (11.2%)	40 (12.0%)	0.683
Sudden cardiac death (*n*, %)	19 (4.1%)	6 (4.8%)	13 (3.9%)	0.385
Death from worsening HF (*n*, %)	35 (7.6%)	8 (6.4%)	27 (8.1%)	0.138
Urgent HTx (*n*, %)	14 (3.1%)	7 (5.6%)	7 (2.1%)	0.002

HF: Heart failure; HTx: Heart transplantation.

**Table 3:** Univariable and multivariable model for the prediction of the composite endpoint.

Total Cohort						
Model	Univariable HR	95% CI	*p*-Value	Multivariable HR	95% CI	*p*-Value
Male sex	1.547	0.791 to 3.026	0.203			
Age	1.002	0.983 to 1.021	0.829			
BMI	0.953	0.897 to 1.013	0.121	0.954	0.887 to 1.027	0.210
LVEF	0.927	0.900 to 0.955	<0.001	0.935	0.905 to 0.966	<0.001
eGFR	0.979	0.969 to 0.989	<0.001	0.986	0.976 to 0.996	0.009
Diabetes	1.196	0.254 to 5.632	0.821			
Smoker	1.716	1.405 to 2.820	0.033	1.395	0.835 to 2.328	0.203
Peak VO_2_	0.835	0.789 to 0.883	<0.001	0.856	0.804 to 0.912	<0.001
Percentage of predicted pVO_2_	0.948	0.934 to 0.963	<0.001	0.955	0.939 to 0.971	<0.001
VE/VCO_2_ slope	1.058	1.041 to 1.075	<0.001	1.064	1.039 to 1.090	<0.001
Peak VO_2_ at AT, mL/kg/min	0.854	0.737 to 0.989	0.035	0.879	0.687 to 1.124	0.305
O_2_ pulse, mL/kg/beat	0.858	0.791 to 0.932	<0.001	0.865	0.780 to 0.961	0.007
Circulatory power, mmHg.mL/kg/min	0.999	0.999 to 0.999	<0.001	0.999	0.998 to 1.000	<0.001
Ventilatory power, mmHg	0.575	0.483 to 0.684	<0.001	0.632	0.521 to 0.768	<0.001
COP	1.118	1.054 to 1.186	<0.001	1.060	0.956 to 1.174	0.268
PetCO_2_ at rest, mmHg	0.887	0.839 to 0.937	<0.001	0.948	0.889 to 1.011	0.102
PetCO_2_ at AT, mmHg	0.862	0.826 to 0.900	<0.001	0.890	0.845 to 0.993	<0.001
**<50 years of age**						
**Model**	**Univariable HR**	**95% CI**	***p*-value**	**Multivariable HR**	**95% CI**	***p*-value**
Male sex	2.172	0.639 to 7.378	0.214			
BMI	0.926	0.835 to 1.027	0.146	0.947	0.810 to 1.107	0.493
LVEF	0.899	0.838 to 0.943	<0.001	0.903	0.835 to 0.976	0.011
eGFR	0.974	0.958 to 0.991	0.002	0.971	0.952 to 0.991	0.005
Diabetes	1.752	0.513 to 5.979	0.371			
Smoker	4.922	1.784 to 13.579	0.002	3.171	1.060 to 9.486	0.039
Peak VO_2_	0.835	0.768 to 0.908	<0.001	0.876	0.793 to 0.968	0.009
Percentage of predicted pVO_2_	0.933	0.907 to 0.961	<0.001	0.951	0.917 to 0.986	0.006
VE/VCO_2_ slope	1.061	1.037 to 1.084	<0.001	1.058	1.016 to 1.101	0.006
Peak VO_2_ at AT, mL/kg/min	0.737	0.559 to 0.971	0.030	0.891	0.691 to 1.091	0.593
O_2_ pulse, mL/kg/beat	0.835	0.724 to 0.962	0.013	0.873	0.711 to 1.071	0.193
Circulatory power, mmHg.mL/kg/min	0.999	0.999 to 0.999	<0.001	0.999	0.999 to 1.000	0.035
Ventilatory power, mmHg	0.514	0.375 to 0.703	<0.001	0.662	0.462 to 0.948	0.024
COP	1.196	1.082 to 1.323	<0.001	1.058	0.954 to 1.162	0.681
PetCO_2_ at rest, mmHg	0.861	0.785 to 0.944	0.001	0.949	0.868 to 1.037	0.949
PetCO_2_ at AT, mmHg	0.871	0.821 to 0.925	<0.001	0.936	0.878 to 0.999	0.047
**≥50 years of age**						
**Model**	**Univariable HR**	**95% CI**	***p*-value**	**Multivariable HR**	**95% CI**	***p*-value**
Male sex	1.338	0.599 to 2.987	0.477			
BMI	0.972	0.901 to 1.048	0.462			
LVEF	0.941	0.908 to 0.974	0.001	0.948	0.913 to 0.984	0.005
eGFR	0.977	0.964 to 0.990	0.001	0.983	0.969 to 0.996	0.013
Diabetes	1.282	0.167 to 2.542	0.474			
Smoker	1.135	0.628 to 2.050	0.675			
Peak VO_2_	0.824	0.765 to 0.887	<0.001	0.823	0.761 to 0.891	<0.001
Percentage of predicted pVO_2_	0.954	0.937 to 0.972	<0.001	0.958	0.939 to 0.977	<0.001
VE/VCO_2_ slope	1.055	1.031 to 1.080	<0.001	1.069	1.038 to 1.101	<0.001
Peak VO_2_ at AT, mL/kg/min	0.961	0.809 to 1.142	0.654			
O_2_ pulse, mL/kg/beat	0.868	0.784 to 0.960	0.006	0.837	0.738 to 0.950	0.006
Circulatory power, mmHg.mL/kg/min	0.999	0.999 to 0.999	<0.001	0.999	0.999 to 0.999	<0.001
Ventilatory power, mmHg	0.601	0.488 to 0.741	<0.001	0.617	0.488 to 0.780	<0.001
COP	1.037	0.941 to 1.142	0.465			
PetCO_2_ at rest, mmHg	0.900	0.839 to 0.964	0.003	0.942	0.869 to 1.022	0.150
PetCO_2_ at AT, mmHg	0.857	0.808 to 0.910	<0.001	0.868	0.810 to 0.931	<0.001

COP: Cardiorespiratory optimal point; LVEF: Left ventricular ejection fraction; eGFR: Estimated glomerular filtration rate; BMI: Body mass index; PetCO_2_: Partial pressure of end-tidal carbon dioxide; AT: Anaerobic threshold; pVO_2_: Peak oxygen consumption; VE/VCO_2_ slope: Minute ventilation–carbon dioxide production ratio.

**Table 4:** ROC curve comparison for the composite endpoint.

	<50 Years (*n* = 125)			≥50 Years (*n* = 333)			
CPET Parameters	AUC	95% CI	*p*-Value	AUC	95% CI	*p*-Value	*p*-Value (Interaction)
pVO_2_, mL/kg/min	0.777	0.669–0.885	<0.001	0.707	0.631–0.784	<0.001	0.311
Predicted pVO_2_ (%)	0.818	0.731–0.904	<0.001	0.703	0.623–0.784	<0.001	0.060
VE/VCO_2_ slope	0.801	0.699–0.902	<0.001	0.696	0.616–0.776	<0.001	0.115
pVO_2_, mL/kg/min at AT	0.723	0.511–0.935	0.018	0.524	0.312–0.735	0.824	0.088
O_2_ pulse, mL/kg/beat	0.656	0.519–0.793	0.024	0.631	0.547–0.716	0.004	0.803
Circulatory power, mmHg.mL/kg/min	0.778	0.663–0.894	<0.001	0.709	0.639–0.780	<0.001	0.399
Ventilatory power, mmHg	0.773	0.646–0.900	<0.001	0.705	0.630–0.780	<0.001	0.373
COP	0.661	0.572–0.750	0.001	0.568	0.479–0.654	0.520	0.168
PetCO_2_ at rest, mmHg	0.711	0.591–0.831	0.003	0.626	0.539–0.712	0.007	0.277
PetCO_2_ at AT, mmHg	0.804	0.706–0.903	<0.001	0.706	0.620–0.792	<0.001	0.145

ROC curve comparison performed with the DeLong et al. test. CPET: Cardiopulmonary exercise testing; COP: Cardiorespiratory optimal point; PetCO_2_: Partial pressure of end-tidal carbon dioxide; AT: Anaerobic threshold; pVO_2_: Peak oxygen consumption; VE/VCO_2_ slope: Minute ventilation–carbon dioxide production ratio.

**Table 5:** Specificity and sensitivity of the cardiopulmonary exercise testing thresholds for the composite endpoint.

	<50 Years (*n* = 125)			≥50 Years (*n* = 333)		
CPET Parameters	Specificity	Sensitivity	Youden Index (*J*)	Specificity	Sensitivity	Youden Index (*J*)
pVO_2_ ≤ 12 mL/kg/min *	98%	29%	0.27	89%	21%	0.10
pVO_2_ ≤ 14 mL/kg/miN	**89%**	**52%**	**0.41**	**77%**	**64%**	**0.41**
VE/VCO_2_ slope >35	80%	62%	0.42	64%	62%	0.26
VE/VCO_2_ slope >32	**72%**	**81%**	**0.53**	54%	81%	0.35
VE/VCO_2_ slope >33	73%	67%	0.40	**58%**	**81%**	**0.39**
Percentage of predicted pVO_2_ ≤ 50%	71%	62%	0.33	83%	47%	0.30
Percentage of predicted pVO_2_ ≤ 56%	65%	81%	0.46	**71%**	**66%**	**0.37**
Percentage of predicted pVO_2_ ≤ 58%	**64%**	**86%**	**0.50**	67%	68%	0.35

* pVO_2_ ≤12 mL/kg/min (or ≤14 mL/kg/min if intolerant to β-blockers). The highest Youden index associated with each CPET parameter is highlighted in bold. CPET: cardiopulmonary exercise testing; pVO_2_: Peak oxygen consumption; VE/VCO_2_ slope: Minute ventilation–carbon dioxide production ratio.

## Data Availability

The data presented in this study are available upon request from the corresponding author. The data are not publicly available due to patient consent regarding availability of individual patient data, applicable only to the local investigation team.

## Supplementary Materials

The following supporting information can be downloaded at: https://www.mdpi.com/article/10.3390/medicina59091685/s1, Figure S1: ROC curves for the composite endpoint in a 36-month follow up. (a) Percentage of predicted pVO_2_ in patients under 50 years old. (b) Percentage of predicted pVO_2_ in patients over 50 years old; Figure S2: Kaplan–Meier survival analysis for the composite endpoint in a 36-month follow-up in patients under 50 years old and patients over 50 years old stratified according to (a) the International Society for Heart and Lung Transplantation (ISHLT) recommended threshold of percentage of predicted peak oxygen consumption (pVO_2_) ≤ 50% and (b) alternative threshold of percentage of predicted pVO_2_ ≤ 58% in patients under 50 years old and percentage of predicted pVO_2_ ≤ 56% in patients over 50 years old.

## References

[1] Corra U., Agostoni P.G., Anker S.D., Coats A.J., Crespo Leiro M.G., de Boer R.A., Harjola V.P., Hill L., Lainscak M., Lund L.H. (2018). Role of cardiopulmonary exercise testing in clinical stratification in heart failure. A position paper from the Committee on Exercise Physiology and Training of the Heart Failure Association of the European Society of Cardiology. Eur. J. Heart Fail..

[2] Chua T.P., Ponikowski P., Harrington D., Anker S.D., Webb-Peploe K., Clark A.L., Poole-Wilson P.A., Coats A.J. (1997). Clinical Correlates and Prognostic Significance of the Ventilatory Response to Exercise in Chronic Heart Failure. J. Am. Coll. Cardiol..

[3] Francis D.P., Shamim W., Davies L.C., Piepoli M.F., Ponikowski P., Anker S.D., Coats A.J.S. (2000). Cardiopulmonary exercise testing for prognosis in chronic heart failure: Continuous and independent prognostic value from VE/VCO_2_ slope and peak VO_2_. Eur. Heart J..

[4] Arena R., Humphrey R. (2002). Comparison of ventilatory expired gas parameters used to predict hospitalization in patients with heart failure. Am. Heart J..

[5] Arena R., Myers J., Aslam S.S., Varughese E.B., Peberdy M.A. (2004). Peak VO_2_ and VE/VCO_2_ slope in patients with heart failure: A prognostic comparison. Am. Heart J..

[6] Kleber F.X., Vietzke G., Wernecke K.D., Bauer U., Opitz C., Wensel R., Sperfeld A., Glaser S. (2000). Impairment of Ventilatory Efficiency in Heart Failure. Circulation.

[7] Mehra M.R., Canter C.E., Hannan M.M., Semigran M.J., Uber P.A., Baran D.A., Danziger-Isakov L., Kirklin J.K., Kirk R., Kushwaha S.S. (2016). The 2016 International Society for Heart Lung Transplantation listing criteria for heart transplantation: A 10-year update. J. Heart Lung Transplant..

[8] Peterson L.R., Schechtman K.B., Ewald G.A., Geltman E.M., De Las Fuentes L., Meyer T., Krekeler P., Moore M.L., Rogers J.G. (2003). Timing of cardiac transplantation in patients with heart failure receiving β-adrenergic blockers. J. Heart Lung Transplant..

[9] Corrà U., Agostoni P., Giordano A., Cattadori G., Battaia E., La Gioia R., Scardovi A.B., Emdin M., Metra M., Sinagra G. (2016). Sex Profile and Risk Assessment with Cardiopulmonary Exercise Testing in Heart Failure: Propensity Score Matching for Sex Selection Bias. Can. J. Cardiol..

[10] Lund L.H., Mancini D.M. (2008). Peak VO_2_ in elderly patients with heart failure. Int. J. Cardiol..

[11] Forman D.E., Clare R., Kitzman D.W., Ellis S.J., Fleg J.L., Chiara T., Fletcher G., Kraus W.E., HF-ACTION Investigators (2009). Relationship of age and exercise performance in patients with heart failure: The HF-ACTION study. Am. Heart J..

[12] Palau P., Domínguez E., Núñez J. (2019). Sex differences on peak oxygen uptake in heart failure. ESC Heart Fail..

[13] Corrà U., Piepoli M.F., Adamopoulos S., Agostoni P., Coats A.J., Conraads V., Lambrinou E., Pieske B., Piotrowicz E., Schmid J.P. (2014). Cardiopulmonary exercise testing in systolic heart failure in 2014: The evolving prognostic role. Eur. J. Heart Fail..

[14] Scardovi A.B., De Maria R., Celestini A., Perna S., Coletta C., Feola M., Aspromonte N., Rosso G.L., Carunchio A., Ferraironi A. (2009). Additive prognostic value of cardiopulmonary exercise testing in elderly patients with heart failure. Clin. Sci..

[15] Arena R., Myers J., Abella J., Pinkstaff S., Brubaker P., Kitzman D.W., Peberdy M.A., Bensimhon D., Chase P., Guazzi M. (2011). Cardiopulmonary exercise testing is equally prognostic in young, middle-aged and older individuals diagnosed with heart failure. Int. J. Cardiol..

[16] Carubelli V., Metra M., Corrà U., Magrì D., Passino C., Lombardi C., Scrutinio D., Correale M., Cattadori G., Piepoli M.F. (2015). Exercise Performance Is a Prognostic Indicator in Elderly Patients with Chronic Heart Failure—Application of Metabolic Exercise Cardiac Kidney Indexes Score. Circ. J..

[17] DeLong E.R., DeLong D.M., Clarke-Pearson D.L. (1988). Comparing the areas under two or more correlated receiver operating characteristic curves: A nonparametric approach. Biometrics.

[18] Kim B.J., Kim Y., Oh J., Jang J., Kang S.M. (2019). Characteristics and Safety of Cardiopulmonary Exercise Testing in Elderly Patients with Cardiovascular Diseases in Korea. Yonsei Med. J..

[19] Sciomer S., Moscucci F., Salvioni E., Marchese G., Bussotti M., Corrà U., Piepoli M.F. (2020). Role of gender, age and BMI in prognosis of heart failure. Eur. J. Prev. Cardiol..

[20] Goldstein D.J., Bello R., Shin J.J., Stevens G., Zolty R., Maybaum S., D’Alessandro D. (2012). Outcomes of cardiac transplantation in septuagenarians. J. Heart Lung Transplant..

[21] Moore B., Brubaker P.H., Stewart K.P., Kitzman D.W. (2007). VE/VCO_2_ Slope in Older Heart Failure Patients with Normal Versus Reduced Ejection Fraction Compared with Age-Matched Healthy Controls. J. Card. Fail..

[22] Mejhert M., Linder-Klingsell E., Edner M., Kahan T., Persson H. (2002). Ventilatory variables are strong prognostic markers in elderly patients with heart failure. Heart.

[23] Cicoira M., Davos C.H., Florea V., Shamim W., Doehner W., Coats A.J., Anker S.D. (2001). Chronic heart failure in the very elderly: Clinical status, survival, and prognostic factors in 188 patients more than 70 years old. Am. Heart J..

[24] Marburger C., Brubaker P., Pollock W., Morgan T., Kitzman D. (1998). Reproducibility of cardiopulmonary exercise testing in elderly patients with congestive heart failure. Am. J. Cardiol..

[25] Koch B., Schäper C., Ittermann T., Spielhagen T., Dörr M., Völzke H., Opitz C.F., Ewert R., Gläser S. (2008). Reference values for cardiopulmonary exercise testing in healthy volunteers: The SHIP study. Eur. Respir. J..

[26] Sun X.G., Hansen J.E., Garatachea N., Storer T.W., Wasserman K. (2002). Ventilatory Efficiency during Exercise in Healthy Subjects. Am. J. Respir. Crit. Care Med..

[27] Salvioni E., Corrà U., Piepoli M., Rovai S., Correale M., Paolillo S., Pasquali M., Magrì D., Vitale G., Fusini L. (2020). Gender and age normalization and ventilation efficiency during exercise in heart failure with reduced ejection fraction. ESC Heart Fail..

[28] Nicholls D., O’Dochartaigh C., Riley M. (2002). Circulatory power—A new perspective on an old friend. Eur. Heart J..

[29] Lala A., Shah K.B., Lanfear D.E., Thibodeau J.T., Palardy M., Ambardekar A.V., McNamara D.M., Taddei-Peters W.C., Baldwin J.T., Jeffries N. (2021). Predictive Value of Cardiopulmonary Exercise Testing Parameters in Ambulatory Advanced Heart Failure. JACC Heart Fail..

[30] Lewis G.D., Zlotoff D.A. (2021). Cardiopulmonary Exercise Testing-Based Risk Stratification in the Modern Era of Advanced Heart Failure Management. JACC Heart Fail..

[31] Agdamag A.C., Van Iterson E.H., Tang W.H.W., Finet J.E. (2023). Prognostic Role of Metabolic Exercise Testing in Heart Failure. J Clin. Med..

[32] Brischetto M.J., Millman R.P., Peterson D.D., Silage D.A., Pack A.I. (1984). Effect of aging on ventilatory response to exercise and CO_2_. J. Appl. Physiol..

[33] Lim Z.X., Gyanwali B., Soh J., Koh A.S., Goh J. (2023). The potential benefits of assessing post-cardiopulmonary exercise testing (CPET) in aging: A narrative review. BMC Sports Sci. Med. Rehabil..

[34] Fortin M., Turgeon P.Y., Nadreau É., Grégoire P., Maltais L.G., Sénéchal M., Provencher S., Maltais F. (2015). Prognostic Value of Oxygen Kinetics During Recovery from Cardiopulmonary Exercise Testing in Patients with Chronic Heart Failure. Can. J. Cardiol..

[35] Sharma A., Verma S., Bhatt D.L., Connelly K.A., Swiggum E., Vaduganathan M., Zieroth S., Butler J. (2022). Optimizing Foundational Therapies in Patients with HFrEF. JACC Basic Transl. Sci..

[36] McDonagh T.A., Metra M., Adamo M., Gardner R.S., Baumbach A., Böhm M., Burri H., Butler J., Čelutkienė J., Chioncel O. (2021). 2021 ESC Guidelines for the diagnosis and treatment of acute and chronic heart failure. Eur. Heart J..

[37] Paolillo S., Veglia F., Salvioni E., Corrà U., Piepoli M., Lagioia R., Limongelli G., Sinagra G., Cattadori G., Scardovi A.B. (2019). Heart failure prognosis over time: How the prognostic role of oxygen consumption and ventilatory efficiency during exercise has changed in the last 20 years. Eur. J. Heart Fail..

[38] McMurray J.J.V., Solomon S.D., Inzucchi S.E., Køber L., Kosiborod M.N., Martinez F.A., Ponikowski P., Sabatine M.S., Anand I.S., Bělohlávek J. (2019). Dapagliflozin in Patients with Heart Failure and Reduced Ejection Fraction. N. Engl. J. Med..

[39] Packer M., Anker S.D., Butler J., Filippatos G., Pocock S.J., Carson P., Januzzi J., Verma S., Tsutsui H., Brueckmann M. (2020). Cardiovascular and Renal Outcomes with Empagliflozin in Heart Failure. N. Engl. J. Med..

[40] McMurray J.J., Packer M., Desai A.S., Gong J., Lefkowitz M.P., Rizkala A.R., Rouleau J.L., Shi V.C., Solomon S.D., Swedberg K. (2014). Angiotensin–Neprilysin Inhibition versus Enalapril in Heart Failure. N. Engl. J. Med..

[41] Gorodeski E.Z., Chu E.C., Chow C.H., Levy W.C., Hsich E., Starling R.C. (2010). Application of the Seattle Heart Failure Model in Ambulatory Patients Presented to an Advanced Heart Failure Therapeutics Committee. Circ. Heart Fail..

[42] Kalogeropoulos A.P., Georgiopoulou V.V., Giamouzis G., Smith A.L., Agha S.A., Waheed S., Laskar S., Puskas J., Dunbar S., Vega D. (2009). Utility of the Seattle Heart Failure Model in Patients with Advanced Heart Failure. J. Am. Coll. Cardiol..

[43] Aaronson K.D., Schwartz J.S., Chen T.M., Wong K.L., Goin J.E., Mancini D.M. (1997). Development and Prospective Validation of a Clinical Index to Predict Survival in Ambulatory Patients Referred for Cardiac Transplant Evaluation. Circulation.

[44] Levy W.C., Arena R., Wagoner L.E., Dardas T., Abraham W.T. (2012). Prognostic Impact of the Addition of Ventilatory Efficiency to the Seattle Heart Failure Model in Patients with Heart Failure. J. Card. Fail..

[45] Agostoni P., Corrà U., Cattadori G., Veglia F., La Gioia R., Scardovi A.B., Emdin M., Metra M., Sinagra G., Limongelli G. (2013). Metabolic exercise test data combined with cardiac and kidney indexes, the MECKI score: A multiparametric approach to heart failure prognosis. Int. J. Cardiol..

[46] Guazzi M., Arena R., Halle M., Piepoli M.F., Myers J., Lavie C.J. (2018). 2016 focused update: Clinical recommendations for cardiopulmonary exercise testing data assessment in specific patient populations. Eur. Heart J..

[47] Malhotra R., Bakken K., D’Elia E., Lewis G.D. (2016). Cardiopulmonary Exercise Testing in Heart Failure. JACC Heart Fail..

[48] Guazzi M., Bandera F., Ozemek C., Systrom D., Arena R. (2017). Cardiopulmonary Exercise Testing. What Is its Value?. J. Am. Coll. Cardiol..

[49] Balady G.J., Arena R., Sietsema K., Myers J., Coke L., Fletcher G.F., Forman D., Franklin B., Guazzi M., Gulati M. (2010). Clinician’s Guide to Cardiopulmonary Exercise Testing in Adults. Circulation.

[50] Arena R., Myers J., Abella J., Peberdy M.A., Bensimhon D., Chase P., Guazzi M. (2007). Development of a Ventilatory Classification System in Patients with Heart Failure. Circulation.

