# Supplementary figures and images for: Age Differences in Cardiopulmonary Exercise Testing Parameters in Heart Failure with Reduced Ejection Fraction
Source: Medicina (Kaunas). 2023 Sep 20;59(9):1685. doi: 10.3390/medicina59091685 (PMC10535443; doi:10.3390/medicina59091685)

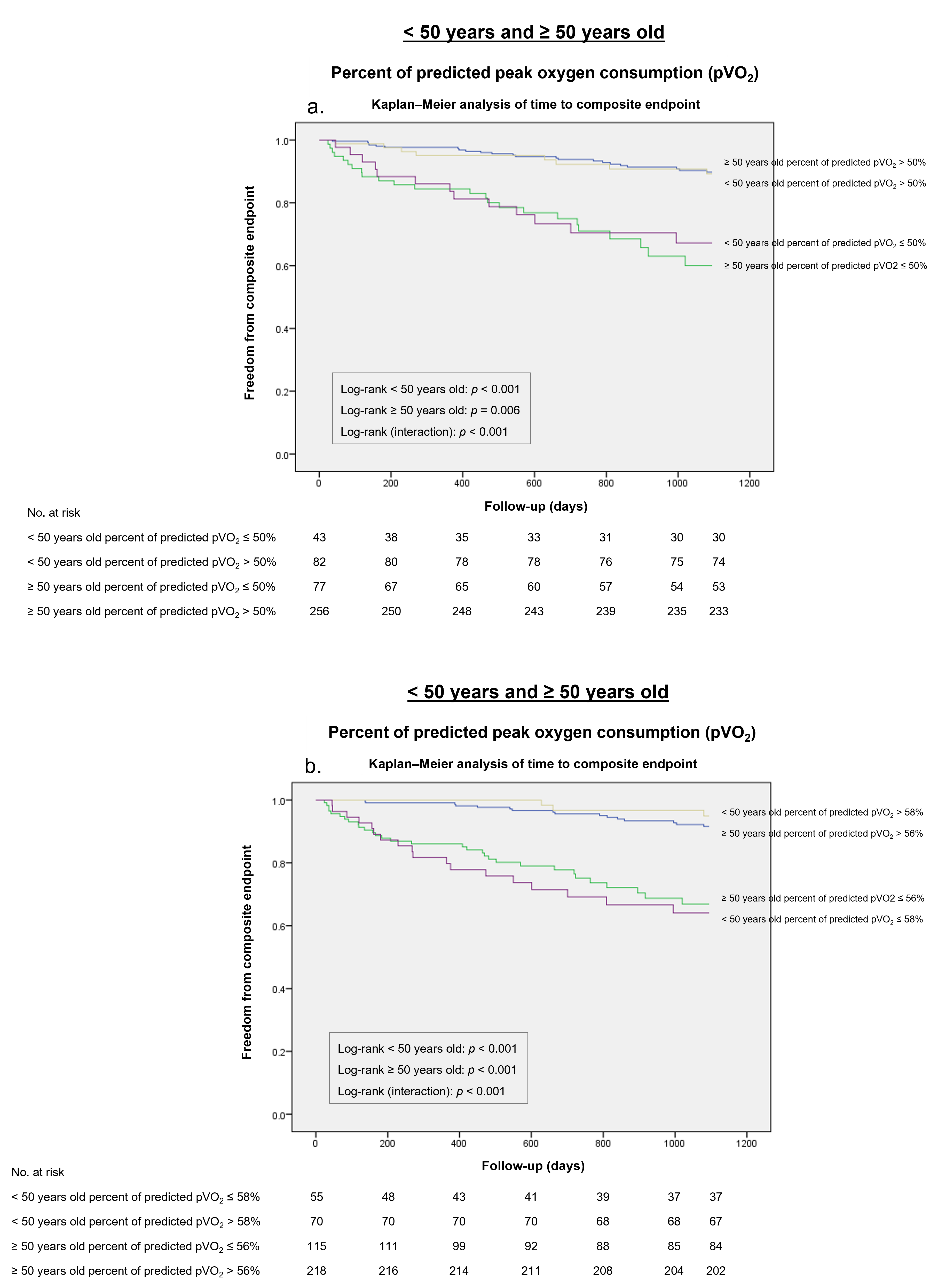

Supplement: Supplementary file 1 [file medicina-59-01685-s001.zip › Supplementary Figure 2.tif]

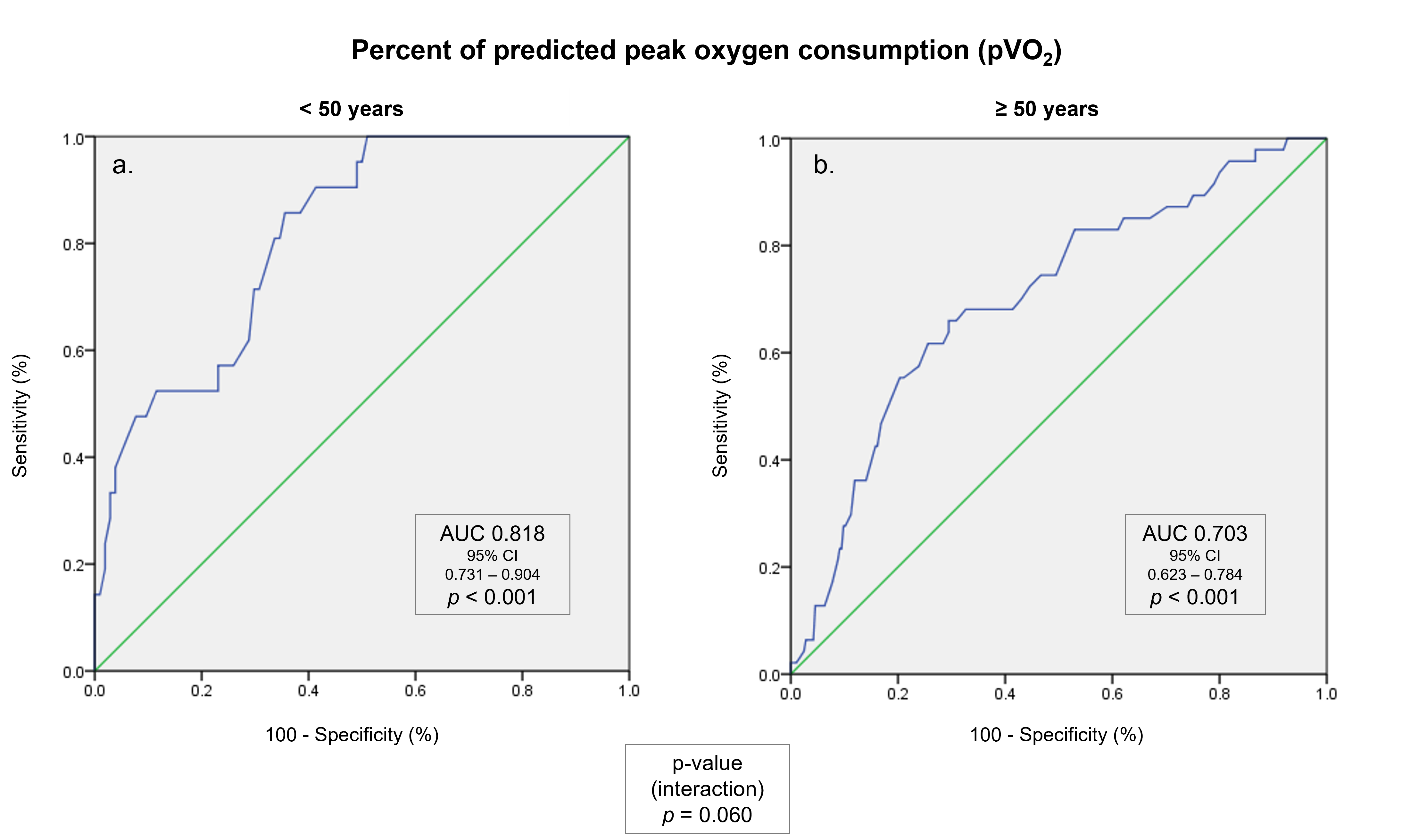

Supplement: Supplementary file 1 [file medicina-59-01685-s001.zip › Supplementary Figure 1.tif]
